# The Floating Forest: Traditional Knowledge and Use of *Matupá* Vegetation Islands by Riverine Peoples of the Central Amazon

**DOI:** 10.1371/journal.pone.0122542

**Published:** 2015-04-02

**Authors:** Carolina T. de Freitas, Glenn H. Shepard, Maria T. F. Piedade

**Affiliations:** 1 Programa de Pós Graduação em Ecologia, Instituto Nacional de Pesquisas da Amazônia, Manaus, Amazonas, Brasil; 2 Departamento de Antropologia, Museu Paraense Emilio Goeldi, Belém, Pará, Brasil; 3 Coordenação de Pesquisas em Dinâmica Ambiental, Instituto Nacional de Pesquisas da Amazônia, Manaus, Amazonas, Brasil; Cirad, FRANCE

## Abstract

*Matupás* are floating vegetation islands found in floodplain lakes of the central Brazilian Amazon. They form initially from the agglomeration of aquatic vegetation, and through time can accumulate a substrate of organic matter sufficient to grow forest patches of several hectares in area and up to 12 m in height. There is little published information on matupás despite their singular characteristics and importance to local fauna and people. In this study we document the traditional ecological knowledge of riverine populations who live near and interact with matupás. We expected that their knowledge, acquired through long term observations and use in different stages of the matupá life cycle, could help clarify various aspects about the ecology and natural history of these islands that field biologists may not have had the opportunity to observe. Research was carried out in five riverine communities of the Amanã Sustainable Development Reserve (Brazil). Semi-structured interviews were conducted with 45 inhabitants in order to register local understandings of how matupás are formed, biotic/abiotic factors related to their occurrence, the plants and animals that occur on them, their ecological relevance, and local uses. Local people elucidated several little-known aspects about matupá ecology, especially regarding the importance of seasonal dynamics of high/low water for matupás formation and the relevance of these islands for fish populations. Soil from matupás is especially fertile and is frequently gathered for use in vegetable gardens. In some cases, crops are planted directly onto matupás, representing an incipient agricultural experiment that was previously undocumented in the Amazon. Matupás are also considered a strategic habitat for fishing, mainly for arapaima (*Arapaima gigas*). The systematic study of traditional ecological knowledge proved to be an important tool for understanding this little-known Amazonian landscape.

## Introduction

**Fig 1:**
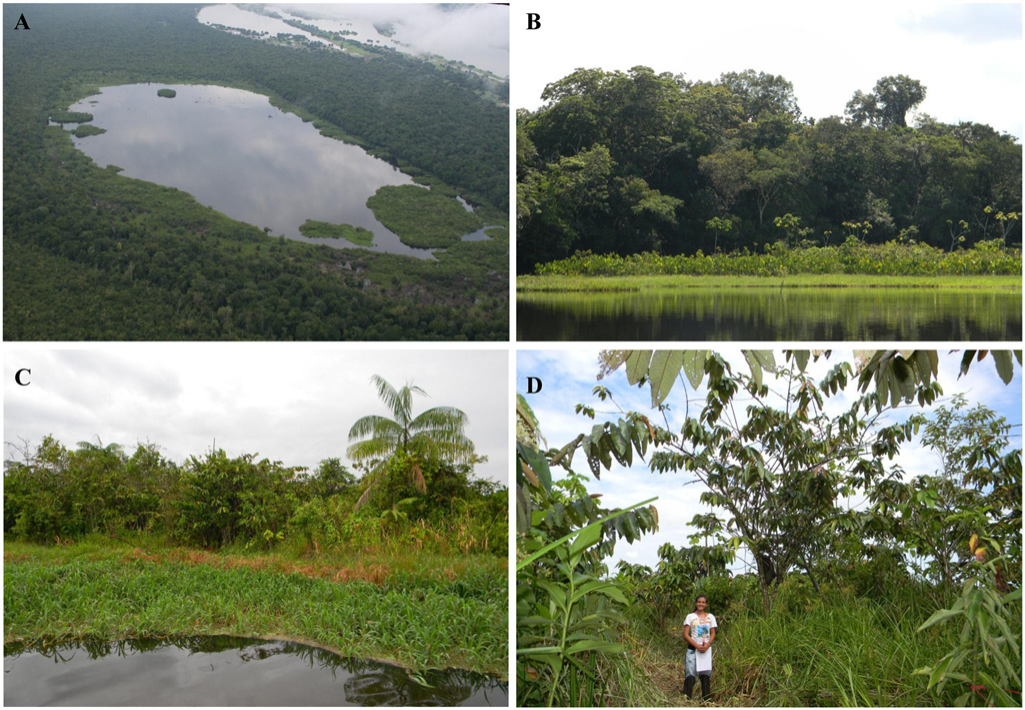
Pictures of matupás in Amanã Sustainable Development Reserve (Amazonas, Brasil). A—Aerial view of matupás floating on a lake; B—Matupá seen from afar, corresponding to the lowest stratum and lighter in color than the forest in the background; C—Matupá seen up close, representing all vegetation in the image; D—CTF on the matupá surface during field work. Photos: A—Florian Wittmann; B-C—Carolina Freitas; D—Divino Áquila Araújo.

Matupá is a local term in the Brazilian Amazon for free-floating islands of vegetation growing on blocks of soil that can be as much as 3 m thick, in sizes ranging from a few square meters to several hectares, and supporting a variety of plant communities from aquatic herbs to shrubs and trees [1,2] (Fig 1). They are found in *várzea* lakes distributed along the floodplains of white-water river systems rich in nutrients and sediments [3]. Matupás are formed through a series of natural successional stages beginning with the agglomeration of aquatic and semi-aquatic plants, and the consequent accumulation of organic substrate where free-standing herbs, shrubs and even trees can later grow [1,2]. Larger, thicker matupás with substantial vegetation are sturdy enough for people to walk on.

Despite anecdotal references in scientific literature to matupás as nesting ground for caimans [4,5] and shelter for manatees [6], there is exceedingly little published information on their ecological characteristics and relevance. Most of what is currently known about matupás is found in just two scientific publications, both concerning flooded environments generally [1,2]. Nonetheless, during exploratory research in the Amanã Sustainable Development Reserve (RDSA; central Brazilian Amazon), we realized that local people made use of matupás both for fishing and agriculture, and appeared to have detailed knowledge about the ecology of these floating forest islands.

Traditional ecological knowledge (see Berkes [7]) can provide insights for studying ecological systems that are important to the livelihoods of local populations [8–15]. Such knowledge accumulates from generation to generation through daily interactions with the environment [7] and can be especially useful for understanding ecological processes that occur at temporal or spatial scales not easily observed by conventional scientific research [16].

Studies of traditional ecological knowledge first emerged within the fields of ethnobotany and ethnobiology, eventually developing into an independent field called ethnoecology [17,18]. Recently, landscape ethnoecology has emerged as a multidisciplinary paradigm for understanding traditional knowledge of the environment at broad spatial and temporal scales [19]. In Amazonia, studies have demonstrated the scientific relevance of traditional landscape ecological knowledge. For example, Shepard *et al*. [10] document habitat classification strategies of the Matsigenka people of Peru, which combine vegetative and geomorphological characters, and suggest how this knowledge could be applied to the interpretation of satellite imagery [16,20]. In a similar but independent study, Fleck and Harder [21] document habitat classification among the Matses people in northeastern Peru; although linguistically and culturally unrelated to the Matsigenka, their classification system likewise combines biotic and abiotic aspects. Moreover, Matses habitat classification accurately predicts vegetation structure, palm species composition and occurrence of small mammals as measured in quantitative studies [21]. Abraão *et al*. [14] compared the ethnoecological classification system of the Baniwa people in the northwest Amazon with botanical surveys and satellite imagery and concluded that indigenous knowledge is an efficient shortcut to assessing habitat diversity [14].

Studies of landscape ethnoecology have shown that local understandings about the environment accurately reflect biotic (e.g. indicator species, forest structure, successional stages) and abiotic factors (e.g. flooding regimes, topography, soil typology) that are significant in local peoples’ relationships with habitats and resources [13,14,16,20,21]. In these aspects, traditional knowledge accurately reflects ecological processes and thus can contribute to scientific studies about the environment at local and regional scales. Yet local knowledge about the environment is also intricately entwined with traditional values, cosmology, spirituality and history, in contrast with scientific worldviews [22–24].

Drawing on these various methodological and theoretical advances, we expected that traditional people living near matupás could help us better understand the ecology of these poorly studied floating islands, especially regarding those aspects that require long-term field observation. By conducting interviews with traditional people of the RDSA, we sought to obtain information about how matupás are formed; what conditions contribute to their occurrence in some places but not others; what plants and animals occur on them; and what uses, ecological function and other significance or importance they have in local peoples’ eyes.

## Methods

### Study Area

**Fig 2:**
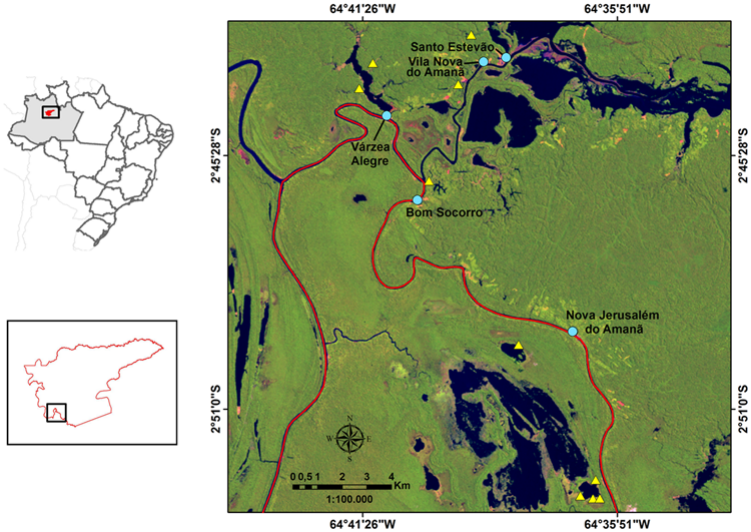
Location of the Amanã Sustainable Development Reserve (RDSA), the communities involved in the study and the matupás inventoried. Upper left: RDSA within Brazil and Amazonas state; lower left: detail of the study region within the reserve; right: LANDSAT 5 image showing the five study communities (blue circles) and the 10 matupás inventoried (yellow triangles); red line indicates reserve border. Adapted from images conceded by USGS Global Visualization Viewer.

The study was carried out within the 2.313.000 ha Amanã Sustainable Development Reserve (Reserva de Desenvolvimento Sustentável Amanã—RDSA; S 02°42', W 64°39') in the central Brazilian Amazon (Fig 2). The reserve, created in 1998, is located along the middle Solimões (Amazon) River near the mouth of the Japurá in Amazonas State. Most of the reserve consists of upland (*terra firme*) forests, but there are also large areas subject to seasonal flooding averaging 10 m difference between minimum and maximum river water level [25].

The reserve is inhabited by about 4000 people living in 26 communities concentrated in the western part of the reserve, mainly along the *várzea* floodplain connected to the Solimões basin by streams and canals. Their economy is based on agriculture, fishing, subsistence hunting, timber extraction and Brazil nut (*Bertholettia excelsa*) gathering. Agriculture is the predominant source of income in the upland *terra firme* communities while fishing predominates in the seasonally flooded *várzea* communities [25].

### Data Collection and Analysis: Interviews

Research on traditional knowledge about matupás was carried out in the communities of Santo Estevão, Vila Nova, Bom Socorro, Nova Jerusalém and Várzea Alegre, all of them located in a *várzea* region in the southwestern portion of the RDSA (Fig 2), and engaged mostly in fishing and agriculture. These communities have between 5 and 30 families, consisting of riverine people or “caboclos” of mixed indigenous, Afro-Brazilian and European ancestry without a marked indigenous ethnic identification [26].

The study communities were chosen after exploratory research in the RDSA, during which we asked local people in several communities where we could find matupás. After identifying and visiting regions where matupás were most common, we chose five communities from which we could most conveniently carry out floristic inventories (see S1 Information for inventories methods). In each study community, we interviewed all adult community members present who agreed to participate. Unfortunately, an extreme flooding event displaced families in some communities and we were unable to conduct interviews with as many people as originally hoped.

We conducted semi-structured interviews [27] in June 2012 with 45 different inhabitants: 32 men and 13 women. The average age of interview subjects was 50 years, with a range of 18 to 78 years old. A total of 35 interview sessions were carried out, of which 28 were with individuals and 7 were with groups of two to three people, usually married couples or members of the same household that were near each other at the moment of the interview. To avoid pseudoreplication, we always consider the total number of interview events (not people interviewed) in the interviews analyses.

All interviews were conducted using the same guideline, covering various aspects including the local definition and classification of matupás, their process of formation, the biotic and abiotic factors related to their occurrence, their botanical and faunal composition, ecological relevance, local uses and other significance (see guideline used in S1 Metadata). One part of the interview included a free-listing activity [28] in which people were asked to name all plants known to exist on matupás. For this activity, subjects were asked about plants known to exist on “matupás in formation” and on “mature matupás”. We chose these two categories based on ongoing interviews and a consensus of local observations regarding the phytophysiognomy distinctive phases during matupá formation.

All interviews were recorded with the subject’s consent and later transcribed, compiled and compared (see secondary data extracted from interviews in S1 Dataset). Especially evocative phrases and comments were transcribed verbatim and are included throughout the text to illustrate local people’s concepts, maintaining their anonymity.

Data from the free-listing exercise were used to calculate the index of cognitive salience [29] for each plant species said to occur on matupás. This index represents the prominence of each plant to local observers across all interviews. The cognitive salience index (*S*) combines the frequency of citation of a given item (*F*) with its mean position across all lists (*mP*): S = F/(N*mP), where *N* is the total number of free-list interviews. *S* varies between 0 and 1, with higher values suggesting greater cognitive salience of a given item during the process of remembering and listing. Plant names mentioned only once were excluded from the analysis.

The cognitive salience index for each woody plant species was compared with results from floristic inventories carried out in October 2012 on 10 matupás located in the study area (Fig 2; see S1 Information for methodological details of inventories). To allow this comparison we used the Importance Value Index (IVI) of the plants inventoried. IVI represents the prominence of each species across all inventories, being calculated by the sum of relative density, relative frequency and relative dominance (basal area) of each species in the inventoried plots [30]. Since in some cases a single local name can refer to more than one botanical species, the IVI was calculated for each species or group of species according to the local name.

### Ethics Statement

The State Center for Protected Areas (Centro Estadual de Unidades de Conservação—CEUC) of the Amazonas State Secretariat of Environment and Sustainable Development (Secretaria de Estado de Meio Ambiente e Desenvolvimento Sustentável do Amazonas—SDS), responsible for the Amanã Sustainable Development Reserve, approved our research project (permit 049/2011) conforming to Brazilian legislation involving scientific research in Protected Areas. The field study did not involve endangered or protected species.

The Human Subjects Research Ethics Committee (Comitê de Ética em Pesquisas com Seres Humanos—CEP) of the Mamirauá Sustainable Development Institute (Instituto de Desenvolvimento Sustentável Mamirauá—IDSM) approved our research protocols (permit 003/2012) conforming to Brazilian legislation involving access to traditional knowledge about biodiversity. In each study community we carried out an initial meeting, explaining our research goals and methods and inviting the families’ voluntary participation in the study. The five communities accepted our research project and all participants signed an Informed Consent Form. At the end of the study, we presented research analyses and results in further meetings with the communities and responded to their questions and observations.

## Results and Discussion

### The concept of matupá

Although the term “matupá” is not well known in the scientific literature nor even among researchers in Amazonia, we found a clear, shared understanding among local people in the study region as to what the term signifies. Matupás were described consistently as floating islands found on lakes, consisting of a thick layer of “soil” (*terra*) covered with dense vegetation. One of those interviewed stated: “The matupá is a forest that floats on the water,” while another said: “In my view, the matupá is water-land [*terra fluvial*]; on top of that water-land rests a forest.” The majority of those interviewed (89%) emphasized the presence of consolidated soil substrate as the necessary condition for floating vegetation to be categorized as a matupá. In this sense, a sharp distinction is made by local people between floating mats of aquatic vegetation and true matupás: the former have no soil substrate, so it is possible to ride over them in a canoe; matupás, by contrast, have a firm, earth-like structure and in many cases are solid enough to walk on. Interview subjects also stated that true matupás harbor a much greater diversity of species than floating mats, including shrubs and trees. This local definition of matupá is very similar to that presented in the available scientific literature, which describes matupás as floating islands with a thick substrate supporting a variety of plant communities from aquatic herbs to shrubs and trees [1,2].

### Formation process

Interview results showed striking similarities in peoples’ descriptions of the formation and development of matupás. Most local people interviewed (89%) cited agglomerations of aquatic or semi-aquatic grasses on lakes, especially *piri* (*Panicum polygonatum* Schrad.) and *membeca* (*Paspalum repens* P.J.Bergius), as the starting point for matupá formation. A majority (71%) pointed out that floating blocks of grasses become inundated with water currents during the flood season and thus “sit down” or “submerge” (*sentam*), sinking to the bottom and dying. During the dry season these blocks of “rotten grasses” (*capins podres*) float back to the surface, providing a substrate of partially decomposed organic matter on which diverse plant species start establishing themselves, initiating a gradual process of succession that can culminate in the growth of trees several years later. The process of “submerging” (*sentar*) and “floating” (*boiar*) of grass blocks often repeats itself several times over the course of successive rainy and dry seasons, marking an essential part in the formation of matupás: “It dies, it lives, dies, lives. And when you think it’s gone, there it is again, fully formed.”

These local observations suggest that matupá formation involves not only natural vegetative succession, as described by Junk [1] and Junk and Piedade [2], but also a crucial early phase determined by seasonal flood dynamics that may be essential to the formation of the matupá substrate, though never before mentioned in the literature. The “sink/float” cycle described by local people should acts as a kind of trampoline, accelerating the otherwise much slower process described by Junk [1] and Junk and Piedade [2] involving agglomeration of aquatic plants and gradual accumulation of sediments and organic material that can last many years. Instead, according to local observations, blocks of semi-rotten grass that sink and float can develop an organic substrate in a matter of months, readily allowing secondary colonization. Furthermore, this “trampoline” might contribute to a greater probability of matupá formation by reducing exposure of the still unformed matupá to disturbances caused by the instability of water conditions throughout the years.

### Environmental conditions and occurrence

Almost all of those interviewed (94%) highlighted the crucial factor of river currents in limiting the formation and occurrence of matupás. Strong currents disrupt the noted processes that contribute to matupá formation. For this reason, matupás are found mostly in lakes, especially those with limited exposure to river currents during the flood season. Some people (40%) noted that matupás usually form along the edges of lakes or slow-moving backwaters (locally known as areas of *ressaca*) where the current is weak. After this period of consolidation and formation in calm waters, matupás may be pushed by wind or currents to other areas of the lake without fragmenting.

A majority of those interviewed (71%) affirmed that matupás occur only in what they call “black water” lakes. According to scientific classifications, however, the lakes studied would not be considered black water systems because of the seasonal flooding by white waters of the Solimões and Japurá rivers. This contradiction owes to different criteria used in local and scientific categories for classifying river systems. The scientific system considers physical and chemical properties and the origin of the water [31] while local people focus on the color itself. When white (i.e., muddy, sediment-laden, non-acidic) waters enter a lake with relatively calm currents, the sediments tend to settle and the water takes on a darker coloration, approximating (visually) that of sediment-free black waters. Thus, what local people call “black water lakes” in this region in fact contain white waters with chemical properties (sediments, higher fertility, higher pH; see [31]) appropriate for the proliferation of aquatic and semi-aquatic plant species that are crucial to matupá formation. Indeed, matupás are found only in the southwestern part of the reserve, corresponding to the *várzea* region which receives yearly influx of white waters from the Solimões and Japurá rivers.

Water depth is another environmental factor noted in about half of interviews (49%): matupás do not occur in lakes that become completely dry and exposed during the dry season. If a matupá settles to the bottom of a dry lake bed or river bank, the plants take root and the vegetation becomes fixed so that when the water rises the matupá “drowns”. Local people say this does not tend to happen with fully developed matupás, because there is always some water under them. This may owe to the fact that to arrive at such an advanced successional stage, a matupá has to be in a lake that does not dry up. However in some interviews (17%) people noted independently the capacity of the matupá to “retain” or “safeguard” (*segurar*) water in a lake. Thus, large matupás may act as a kind of sponge retaining water such that, even if the lake were to dry out during an extreme drought, the matupá substrate retains a layer of water between itself and the lake bottom.

### Biotic aspects of matupás: flora

The cognitive salience index of each plant cited in interviews was calculated separately for the stages of “matupá in formation” and “mature matupá”. The majority of those interviewed (57%) cited only woody plants for mature matupás, with the exception of the arborescent *aninga* (*Montrichardia linifera* (Arruda) Schott) which though technically an herbaceous plant, can grow up to 6 meters in height. The tendency to associate herbaceous plants with matupás in formation and woody plants with mature matupás probably owes to the complete transformation in phytophysiognomy these islands undergo during succession and development. In their early stages, matupás are loosely structured mats of herbaceous plants, while in later stages they resemble small forests, with many shrubs and trees on a solid surface. Nonetheless when asked in interviews, after the free-listing exercise was over, if there were also herbs on mature matupás many people stated that some herbaceous plants remain.

For the matupá in formation, *piri* (*Panicum polygonatum*) had the highest cognitive salience index (S = 0.54; Table 1), followed by *capim navalha* (*Rhynchospora corymbosa* (L.) Britton; S = 0.23; Table 1) and *membeca* (*Paspalum repens*; S = 0.19; Table 1). The other plants cited had a cognitive salience index smaller than 0.13 (Table 1). For the mature matupá, *lacre* (*Vismia* spp.) was the plant with the highest cognitive salience index (*S* = 0.36; Table 2), followed by *açaí* (*Euterpe precatoria* Mart.; S = 0.29; Table 2), *aninga* (*Montrichardia linifera*; S = 0.25; Table 2), *apuí* (referring to both *Ficus* spp. and *Clusia* spp.; S = 0.16; Table 2) and *embaúba* (*Cecropia* spp.; S = 0.16; Table 2). Other plants cited had a cognitive salience index lower than 0.09 (Table 2).

**Table 1:** Frequency of citation, mean citation position and cognitive salience index of each plant associated with the “matupás in formation” by inhabitants of the Amanã Sustainable Development Reserve (Amazonas, Brazil).

Plant	Frequency of citation	Mean citation position	Cognitive salience index
Piri (*Panicum polygonatum* Schrad.)	18	1.39	0.54
Capim navalha (*Rhynchospora corymbosa* (L.) Britton)	11	2.00	0.23
Membeca (*Paspalum repens* P.J.Bergius)	07	1.57	0.19
Batatarana (*Ipomoea aquatica* Forssk.)	07	2.29	0.13
Aninga (*Montrichardia linifera* (Arruda) Schott)	07	3.43	0.09
Rabo de cavalo (*Hymenachne amplexicaulis* (Rudge) Nees)	04	2.50	0.07
Mureru^a^	03	3.33	0.04
Canarana (*Echinochloa polystachya* (Kunth) Hitchc.)	02	2.50	0.03
Urtiga (*Laportea aestuans* (L.) Chew)	02	3.00	0.03
Capim arroz (*Oryza grandiglumis* (Döll) Prodoehl)	02	4.50	0.02

The cognitive salience index (*S*) is calculated according to the formula: S = F/(N*P_*m*_ *)*, in which *F* = frequency of citation; *P*_*m*_ = mean position (rank) of the term in individuals lists; and *N* = total number of lists (interviews). Total number of lists for the “matupás in formation” = 25 (some of the interviewed did not consider “matupá in formation” to be a true kind of matupá).

^a^ *Mureru* is a generic term used to name several floating herbaceous species, especially those that do not extend itself above the water surface, keeping its leaves on the water surface (e.g.: *Pistia stratiotes*, *Hydrocotyle ranunculoides*, *Azolla caroliniana*, *Azolla microphylla*, *Limnobium spongia*, *Ludwigia helminthorrhiza*, *Phyllanthus fluitans*, *Pontederia rotundifolia*, *Eichhornia crassipes*, *Salvinia minima*).

**Table 2:** Frequency of citation, mean citation position, cognitive salience index and Importance Value Index (IVI) of each plant associated with the “mature matupás” by inhabitants of the Amanã Sustainable Development Reserve (Amazonas, Brazil).

Plant	Frequency of citation	Mean citation position	Cognitive salience index	IVI^a^
Lacre (*Vismia* spp.)	30	2.40	0.36	58.77
Açaí (*Euterpe precatoria* Mart)	33	3.24	0.29	37.62
Aninga (*Montrichardia linifera* (Arruda) Schott)	19	2.16	0.25	—
Apuí (*Ficus* spp. and *Clusia* spp.)	20	3.50	0.16	83.17
Embaúba (*Cecropia* spp.)	21	3.76	0.16	8.03
Munguba (*Pseudobombax munguba* (Mart. & Zucc.) Dugand)	14	4.50	0.09	16.06
Capim navalha (*Rhynchospora corymbosa* (L.) Britton)	09	3.78	0.07	—
Caxinguba (*Ficus maxima* Mill.)	10	4.20	0.07	9.31
Piri (*Panicum polygonatum* Schrad.)	10	4.60	0.06	—
Matapasto (*Chromolaena maximilianii* (Schrad. Ex DC.) R.M.King & H.Rob.)	04	3.50	0.03	0.00
Tachi (*Triplaris surinamensis* Cham.)	04	4.50	0.03	31.55
Rabo de camaleão (*Acacia loretensis* J.F.Macbr.)	02	3.00	0.02	1.53
Jacareúba (*Calophyllum brasiliense* Cambess.)	02	3.50	0.02	5.18
Jauari (*Astrocaryum jauari* Mart.)	03	5.67	0.02	0.00
Rabo de cavalo (*Hymenachne amplexicaulis* (Rudge) Nees)	03	7.00	0.01	—
Canafiche (*Costus arabicus* L.)	02	5.50	0.01	—
Piranheira (*Piranhea trifoliata* Baill.)	02	6.50	0.01	0.00

The cognitive salience index (*S*) is calculated according to the formula: S = F/(N*P_*m*_ *)*, in which *F* = frequency of citation; *P*_*m*_ = mean position (rank) of the term in individuals lists; and *N* = total number of lists (interviews). Total number of lists for the “mature matupás” = 35.

^a^ IVI was obtained from inventories conducted in 10 matupás located in the study area (see S1 Information for details about inventories). IVI is calculated by the sum of relative density, relative frequency and relative dominance (basal area) of each woody plant species across all inventoried plots. Here, IVI was calculated for each species or group of species according to the local name.

The three most salient woody plants mentioned in interviews (*lacre*, *açaí* and *apuí*) were also the most prominent species in the floristic inventories (Table 2; see also S1 Table for details about all species registered in the inventories). *Embaúba* was not registered as often in our floristic inventories but is mentioned by Junk [1] and Junk and Piedade [2] in their observations of common tree species on matupás. *Munguba* (*Pseudobombax munguba* (Mart. & Zucc.) Dugand) and *caxinguba* (*Ficus maxima* Mill.) were more salient in the interviews than *tachi* (*Triplaris surinamensis* Cham.), even though the latter was more prominent in inventories (Table 2). However the former two species (*munguba*, *caxinguba*) were present in more matupás inventoried than the latter (S1 Table), suggesting that local perceptions of plant salience are based on holistic assessments, including not only the local abundance but also the frequency of occurrence of each plant in multiple examples of the same habitat.

### Biotic aspects of matupás: fauna

The people of Amanã consider matupás to be places harboring many animal species, both on top of the vegetation island as well as in the water immediately around and below. The most frequently mentioned animals were caimans (*Caiman crocodilus* and *Melanosuchus niger*); yellow-headed side-neck turtle (*Podocnemis unifilis*); Amazonian manatee (*Trichechus inunguis*); fish, especially arapaima (*Arapaima gigas*) and tambaqui (*Colossoma macropomum*); capybara (*Hydrochoerus hydrochaeris*); jaguar and puma (*Panthera onca*, *Puma concolor)*; snakes; birds, especially the wattled jacana (*Jacana jacana*), hoatzin (*Opisthocomus hoazin*) and horned screamer (*Anhima cornuta*); and insects (Table 3).

**Table 3:** Percentage of interviews in which each animal was said to occur in and around matupás in two stages of growth in the Amanã Sustainable Development Reserve (Amazonas, Brazil).

Local name			Frequency of citation	
	English name	Species	*Matupá in formation*	*Mature matupá*
Jacaré	caiman	*Caiman crocodilus*, *Melanosuchus niger*	72% (18)	86% (30)
Cobras	(snakes)		64% (16)	80% (28)
Tracajá	yellow-headed side-neck turtle	*Podocnemis unifilis*	60% (15)	71% (25)
Capivara	capybara	*Hydrochoerus hydrochaeris*	28% (7)	66% (23)
Onça	jaguar, puma	*Panthera onca*, *Puma concolor*	8% (2)	49% (17)
Peixe-boi	Amazonian manatee	*Trichechus inunguis*	52% (13)	46% (16)
Insetos	(insects)		60% (15)	43% (15)
Pirarucu	arapaima, pirarucu	*Arapaima gigas*	36% (9)	43% (15)
Tambaqui	tambaqui	*Colossoma macropomum*	32% (8)	43% (15)
Peixes^a^	(fish [generic])		28% (7)	26% (9)
Pássaros	(birds)		28% (7)	26% (9)
Aranhas	(spiders)		12% (3)	9% (3)
Rato	(rat, mouse)		20% (5)	6% (2)
Sapos	(frogs, toads)		8% (2)	3% (1)

Actual number of interviews is included in parentheses. For the “matupá in formation” the total number of interviews is 25, while for the “mature matupá” is 35 (some of the interviewed did not consider “matupá in formation” to be a true kind of matupá). Percent values are rounded to nearest whole number, presented in order of decreasing frequency for mature matupás.

^a^ Generic mentions of unspecified “fish”, excluding specific mentions of important species like *pirarucu* and *tambaqui*.

There is very little information in the scientific literature about the use of matupás by animal species. In a review of the Amazonian manatee, Pereira [6] mentions that this species shows a high preference for matupás as an ecological zone providing shelter. Hoogmoed [4] and Villamarín *et al*. [5] have mentioned the use of matupás as nesting grounds by *C*. *crocodilus* and *M*. *niger*, respectively. Villamarín *et al*. [5] suggest that caimans use matupás for nesting because the eggs are protected from flooding, as the mature islands remain always afloat. Additional studies on the use of matupás by animal species are necessary and could provide important information about the relationship between this special habitat and the local fauna.

### Ecological importance of matupás

When questioned about the ecological significance of matupás, all of those interviewed (100%) argued that these floating islands are important for aquatic life, especially for large fish species like arapaima (*pirarucu*), tambaqui and the Amazonian manatee (note that local people classify the manatee, an aquatic mammal, within the category of “large fish”; [32]). Lakes with matupás are said to harbor larger populations of these species than lakes without matupás. One of those interviewed, for example, noted: “The matupá is the house of the fish. A lake with lots of matupás is a lake that concentrates lots of fish.” Another stated:

*The matupá is part of preservation*. *You can see that if a lake doesn’t have a matupá*, *the fish leave right away*. *When there’s a matupá*, *the fish stay there*. *So matupás are really important*. *There is no other place with so many fish as where there are matupás*.

Local people attributed the greater abundance of fish and other aquatic animals to the matupá’s functioning as a kind of “safe house” (*abrigo*), attracting and sustaining animal populations and providing them with protection, food and more stable temperatures. Some (26%) see matupás as contributing indirectly to fish abundance by creating cover and protecting them from excessive fishing.

Although aquatic vegetation mats are recognized as providing shelter, food and nesting grounds for many fish species [33–35], the current scientific literature contains no specific reference to the ecological importance of fully formed matupás for fish populations. Nevertheless, with their thick layer of partially decomposed organic matter, matupás must provide natural cover and good shelter for fish. Matupás also appear to create micro-habitats near the water surface and contribute plentiful biological material to nourish diverse species.

In some interviews (34%) local people highlighted the importance of matupás in providing shelter and nesting grounds for other aquatic animals, especially caimans, turtles and birds. As noted above, this fact was registered for caiman species by Hoogmoed [4] and Villamarín *et al*. [5].

The relevance of matupás for plant species, providing a fertile substrate for germination and growth, was also cited in a few interviews (9%). In others (17%) people noted a role for the matupá in conserving the water of the lake, including stories about lakes that dried up after a matupá was removed. These observations are associated with the idea of the matupá acting as a kind of “sponge,” discussed above.

### Importance of matupás for riverine populations: local uses

In 89% of the interviews, inhabitants of RDSA pointed out the value of soil from matupás as a source of fertilizer. They stated that matupá soil provides excellent natural fertilizer for onion (*Allium cepa* L.), chives (*Allium fistulosum* L.), peppers (*Capsicum* sp.) and other spices grown mostly in raised beds by local people. One woman noted emphatically:

*Ah*! *I always use matupá*, *the vegetables come up so pretty… My husband’s sister-in-law made a little garden over there and used only matupá*. *Go have a look to see how pretty her peppers are*! *So beautiful*. *Beautiful*, *beautiful*. *Only with matupá*!

Matupá soil is gathered in the dry season, usually taken from matupás that are still in the early process of formation. As lake levels drop during the dry season these incipient matupás, that already have a layer of organic matter but no large plants, often “land” (*ficar em terra*) which is to say, settle along the lake margin and begin to dry out. These “dead” matupás are the ones most often used by the people as fertilizer. For some people getting to matupás during the dry season can be difficult, but those who have access use them frequently, if not exclusively, to fertilize their vegetables and spices.

Junk [36] had noted the potential use of matupás as an effective and practical fertilizer. Noda *et al*. [37] carried out a series of experiments to test the fertilizing potential of matupá compared to other natural fertilizers on plantations of winged bean (*Psophocarpus tetragonolobus* (L.) DC.), and concluded that matupá is more effective than all other natural fertilizers tested including chicken manure and compost from fruit and vegetable remains. Although such dry blocks are sometimes involved in matupá formation (see above), the amount of soil extracted is minimal compared to the number of blocks of rotten grass that float to the surface during the dry season. With current levels and means of extraction, local use of matupá soil for fertilizer does not appear to endanger the persistence or formation of matupás in the region.

Six people interviewed (17%) claimed to have planted crops directly onto matupás, a phenomenon that has never been mentioned in the scientific literature. These six people stated that they had planted on the same kind of dried-out, “dead” matupás that are commonly used for gathering garden fertilizer. According to them, crops planted include maize (*Zea mays* L.), watermelon (*Citrullus lanatus* (Thunb.) Matsum. & Nakai) and/or bur cucumber (‘*maxixe*’; *Cucumis anguria* L.). Interviewees also mentioned the names of three other local inhabitants who had planted crops on matupás in the past, but these people were no longer in the region to be interviewed. Upon consulting researchers working in other regions of Brazilian Amazon, a few similar cases of agricultural production on matupás were registered for example in the Piagaçu-Purus Sustainable Development Reserve (José Rabello Neto, personal communication) and in Barrerinha municipality (Maria N.F. da Silva, personal communication). However, both in Amanã and in these other regions, all such cases seemed to be the result of experimentation by a few individuals rather than a consistent or widespread agricultural strategy.

The cultivation on “dead” matupás located on the lake shore involves a serious risk of flooding, limiting more widespread use, because even though the seasonal flood cycle in the region is highly predictable [38], the lakes where matupás occur are vulnerable to sudden flash-floods (*repiquetes*). Heavy rain storms can also cause water levels to rise especially in smaller lakes. On the other hand, fully formed, floating matupás are not typically used for agriculture because local people say that these matupás would have to be cleared and burned to support crops; since they have a plentiful supply of more easily accessible *terra firme* forests for agriculture, they do not use matupás in this way.

In 60% of the interviews people pointed out the importance of matupás for fishing. As noted above, matupás are thought to support a greater abundance of aquatic life than in lakes without matupás. Matupás are also considered a strategic place for fishing, especially for the highly valued arapaima (*Arapaima gigas*). Fishermen claim that arapaima often hides under the matupá and comes up for air—the species breathes atmospheric oxygen—in protected holes within the island. Some of those interviewed (14%) stated that the arapaima itself creates these holes by clearing away substrate during the early stages of matupá formation, and then maintains the opening as the matupá thickens and matures. Somewhat analogous to Inuit hunters who wait by holes in the ice to harpoon seals, local fisherman clear a trail to air holes in the matupá to harpoon the arapaima when it comes up to breathe. Although only one of those interviewed claimed to have done so personally, 18 others mentioned cases of fisherman they knew who had hunted arapaima on matupás in the past. With the fairly recent prohibition of arapaima capture in the absence of formalized management plans [39,40], local people appear somewhat reticent about discussing this activity.

Some people (23%) mentioned gathering turtle and caiman eggs, considered local delicacies, on matupás. This activity appears to be more opportunistic than systematic, with people stopping occasionally to gather eggs on a matupá where they observe a nest from a distance.

### Matupás and cosmology: the legend of the Giant Anaconda

During interviews and other research activities, many people noted the association of matupás with the legendary *cobra grande* or “Giant Anaconda”, a widespread element of Amazonian folklore that has been related in some accounts to various natural phenomena [41–43]. Local people consider the Giant Anaconda to be the “mother of the lakes” or the “mother of the waters,” an important being who acts as a guardian of aquatic life and water itself. For them, the Giant Anaconda is no legend but rather a real and terrifying creature, a gigantic anaconda-like snake that can make the surface of the lake tremble, echo with explosive percussions and unleash severe thunderstorms. As one person stated: “She brings thunderstorms. Anytime there is a strong thunderstorm, it’s because that snake has floated to the surface somewhere nearby.”

In 51% of the interviews, people stated that the matupá is a place used by the Giant Anaconda for shelter. People often refer to the matupá as the “house of the Giant Anaconda,” and some (14%) claimed that this creature is essential to the very formation of matupás. One person stated: “We think the Snake’s got a magnet to make the matupá grow where she lives.” Another said:

*Matupás only form where there’s a Snake*: *that’s its mother*. *If there is no mother*, *no matupá can form… If the mother dies*, *the matupá is finished*. *It’s not just any snake*, *it has to be the Giant Anaconda*. *They say it’s not true*, *but it is*. *It exists*.

Some (11%) claimed that the Giant Anaconda ensures that matupás never fully dry out. Only when the Giant Anaconda leaves the lake does the matupá dry up and the lake empty out. The bigger the matupá, the higher the probability a Giant Anaconda lives under it, and hence the greater sense of fear and respect towards that matupá:

*That’s the place where she stays*, *it’s her house*, *that’s why she makes the matupá*. *My grandfather told me this*. *He said that where there’s a Snake*, *there’s a matupá that grows really big*, *because that’s her house*.

Though belief in the Giant Anaconda is not universal among inhabitants of the region, it is certainly widespread and even dominant in local tradition. Denial of the Giant Anaconda is found more in the younger generations, some of whom state that the Giant Anaconda is an “old people’s tale”. And yet the tradition is so deeply ingrained that even those who claim not to believe in the legend show the same sense of timidity and respect towards matupás, often steering clear of them or of lakes where large matupás are present.

Thus, matupás have a cosmological importance beyond their direct and indirect value to local people. The association of matupás with the Giant Anaconda may contribute to the conservation of these islands and associated fauna and flora, since local people respect and somewhat fear larger matupás and avoid visiting them due to the presence of this mythical creature.

## Conclusions

Riverine people in Amanã Sustainable Development Reserve have detailed knowledge about the matupás and value them for practical purposes, especially agriculture and fishing. These people elucidated certain aspects about matupá ecology that had not yet been mentioned in the scientific literature, especially about the formation process of matupás and the importance of these islands for fish populations. Indeed, some of the information obtained in interviews would have taken years of field observation to be detected by researchers. A growing number of studies have documented local and indigenous knowledge about the environment and pointed out its scientific value, but there remain serious barriers of communication, appreciation and respect among mainstream biologists towards traditional ecological knowledge [9,12]. Our study contributes to these efforts by verifying certain local observations about matupá composition, successional dynamics and ecological functions, while also pointing out aspects so far overlooked in the scientific literature and suggesting avenues for further research. Yet local beliefs associating matupás with the legend of the Giant Anaconda reinforce the way traditional conceptions of landscape are inextricably linked with cosmology and cultural values that go beyond practical concerns. Landscape ethnoecology brings traditional knowledge into dialog with scientific understandings, providing novel observations, complementary perspectives and alternative views on the relationship between humans and the environment.

## Supporting Information

S1 Dataset Secondary data extracted from 35 interviews conducted with inhabitants of the Amanã Sustainable Development Reserve (Amazonas, Brazil). (XLSX)

S1 Information Methods of floristic inventories conducted in 10 matupás located in and around the Amanã Sustainable Development Reserve (Amazonas, Brazil). (DOCX)

S1 Metadata Guideline used to conduct interviews with inhabitants of the Amanã Sustainable Development Reserve (Amazonas, Brazil). (DOCX)

S1 Table Number of individuals, number of matupás where the species occurred, relative density, relative frequency, relative dominance and Importance Value Index (IVI) of each woody species registered in the 10 matupás inventoried in and around the Amanã Sustainable Development Reserve (Amazonas, Brazil). (DOCX)
