# Supplementary material for: The Floating Forest: Traditional Knowledge and Use of Matupá Vegetation Islands by Riverine Peoples of the Central Amazon
Source: PLoS One. 2015 Apr 2;10(4):e0122542. doi: 10.1371/journal.pone.0122542 (PMC4383509; doi:10.1371/journal.pone.0122542)
Supplement: S1 Information — (DOCX) [file pone.0122542.s002.docx]

**Methods of floristic inventories**

We inventoried 10 matupás located in six different bodies of water, all of them situated in the *várzea* region of the southwestern portion of RDSA, including lakes along the reserve border (Figure 2). Matupás were chosen according to accessibility and viability of walking on the matupá surface to carry out the survey. Only mature matupás were inventoried because matupás in early phases of formation are not firm enough to permit such inventories. All inventories were conducted in October 2012, which corresponds to a dry period in the study area.

In each matupá we established two central perpendicular trails, each one on the longer axis of the matupá in each direction. To collect floristic data we established, tangent to these two trails, plots of 5 X 5m every 30 m alternating between the right and left side of the trail. The number of plots per matupá inventoried ranged from five to 14, depending on size; in the majority of matupás (70%) at least seven plots were sampled. In total, we sampled 82 plots.

In each plot we countered all shrubs and trees that had a Diameter at Breast Heigh (DBH) ≥ 5 cm and all palms that had stipe ≥ 1 m. We registered the DBH and the height of each individual sampled by using a diameter tape and a measuring pole respectively. All morphospecies were collected for later botanical identification by consulting botanical guides, specialists and herbarium collection. Vouchers collected were deposited in the herbarium of the Instituto Federal de Educação, Ciência e Tecnologia do Amazonas (Herbário EAFM/IFAM), located in Manaus (Amazonas, Brasil). In total, we registered 170 individuals of 28 species, from 23 genera and 17 botanical families (S1 Table).
