# Supplementary material for: The Floating Forest: Traditional Knowledge and Use of Matupá Vegetation Islands by Riverine Peoples of the Central Amazon
Source: PLoS One. 2015 Apr 2;10(4):e0122542. doi: 10.1371/journal.pone.0122542 (PMC4383509; doi:10.1371/journal.pone.0122542)
Supplement: S1 Metadata — (DOCX) [file pone.0122542.s003.docx]

**Guideline for interviews**

Date:

General information about the interviewees

1. Name:
2. Age/Sex:
3. In which community do you live? How long?
4. Did your ancestors live there?
5. Where did your ancestors come from?
6. What are the sources of income of your family?

Information about the concept of matupás, their characteristics, their formation process and factors related to their occurrence

7. What is a matupá?

8. How are matupás formed?

9. What must have or cannot have (biotic/abiotic factors) to allow the formation of matupás in a certain place?

- Color of water:
- Water current:
- Lake depth:
- Lake depth range (flood season x dry season):
- Lake format:
- Lake area:
- Distance to the river:
- Connectivity with the river:
- Animals presence:
- Plants presence:
- Other:

1. Are there different types (or stages) of matupás? Which ones? How do you distinguish them?

| Type |  |  |
| --- | --- | --- |
| Stage |  |  |
| Time for formation |  |  |
| Size |  |  |
| Substrate depth |  |  |
| Substrate appearance |  |  |
| Plant species |  |  |
| Animal species |  |  |
| Other |  |  |

Information about the importance and local use of matupás

1. Do matupás have any importance for nature? Which one?
2. Can matupás be useful for people who live here in the reserve? How?
3. Do you make or have made some kind of use of matupá? Which one? How often?
4. What are the advantages and disadvantages of this use of matupás?
5. Do you think that this use of matupás have influence on the development and integrity of matupás? Which one?
