# Supplementary material for: The Floating Forest: Traditional Knowledge and Use of Matupá Vegetation Islands by Riverine Peoples of the Central Amazon
Source: PLoS One. 2015 Apr 2;10(4):e0122542. doi: 10.1371/journal.pone.0122542 (PMC4383509; doi:10.1371/journal.pone.0122542)
Supplement: S1 Table — (DOCX) [file pone.0122542.s004.docx]

| **S1 Table.** Number of individuals, number of matupás where the species occurred, relative density, relative frequency, relative dominance and Importance Value Index (IVI) of each woody species registered in the 10 matupás inventoried in and around the Amanã Sustainable Development Reserve (Amazonas, Brazil). | | | | | | |
| --- | --- | --- | --- | --- | --- | --- |
| **Botanical family**  Species | Number of individuals | Number of matupás where the species occurred | Relative density (%) | Relative frequency (%) | Relative dominance (%) | IVI |
| **Apocynaceae**  *Himatanthus sucuuba* (Spruce ex Müll.Arg.) Woodson | 1 | 1 | 0,6 | 0,8 | 0,2 | 1,7 |
| **Arecaceae**  *Euterpe precatoria* Mart. | 17 | 6 | 10,0 | 10,2 | 15,9 | 36,0 |
| **Calophyllaceae**  *Calophyllum brasiliense* Cambess. | 3 | 3 | 1,8 | 2,5 | 0,6 | 4,9 |
| **Clusiaceae**  *Clusia* cf. *panapanari* (Aubl.) Choisy | 39 | 6 | 22,9 | 16,9 | 14,6 | 54,5 |
| **Euphorbiaceae**  *Alchornea discolor* Poepp. | 1 | 1 | 0,6 | 0,8 | 0,2 | 1,6 |
| *Glycydendron amazonicum* Ducke | 1 | 1 | 0,6 | 0,8 | 2,3 | 3,7 |
| *Sapium glandulosum* (L.) Morong | 1 | 1 | 0,6 | 0,8 | 1,1 | 2,5 |
| **Fabaceae**  *Hydrochorea corymbosa* (Rich.) Barneby & J.W.Grimes | 3 | 2 | 1,8 | 2,5 | 1,1 | 5,4 |
| *Inga acreana* Harms | 1 | 1 | 0,6 | 0,8 | 0,2 | 1,6 |
| *Macrolobium acaciifolium* (Benth.) Benth. | 4 | 3 | 2,4 | 2,5 | 7,4 | 12,3 |
| *Acacia loretensis* J.F.Macbr. | 1 | 1 | 0,6 | 0,8 | 0,0 | 1,4 |
| **Hypericaceae**  *Vismia japurensis* Rchb.f. | 7 | 3 | 4,1 | 5,1 | 2,7 | 11,9 |
| *Vismia sandwithii* Ewan | 32 | 8 | 18,8 | 15,3 | 13,7 | 47,8 |
| **Lauraceae**  *Nectandra amazonum* Nees | 1 | 1 | 0,6 | 0,8 | 0,3 | 1,8 |
| **Malpighiaceae**  *Byrsonima japurensis* A.Juss. | 1 | 1 | 0,6 | 0,8 | 0,6 | 2,0 |
| **Malvaceae**  *Pseudobombax munguba* (Mart. & Zucc.) Dugand | 9 | 5 | 5,3 | 5,9 | 4,0 | 15,2 |
| **Melastomataceae**  *Miconia* sp. | 1 | 1 | 0,6 | 0,8 | 0,2 | 1,6 |
| **Moraceae**  *Ficus* cf. *gomelleira* Kunth & C.D.Bouché | 2 | 1 | 1,2 | 1,7 | 1,0 | 3,8 |
| *Ficus* cf. *mathewsii* (Miq.) Miq. | 3 | 3 | 1,8 | 2,5 | 1,9 | 6,3 |
| *Ficus maxima* Mill. | 5 | 4 | 2,9 | 3,4 | 2,6 | 8,9 |
| *Ficus* sp. | 12 | 4 | 7,1 | 7,6 | 7,9 | 22,6 |
| *Ficus* sp.2 | 1 | 1 | 0,6 | 0,8 | 0,7 | 2,2 |
| **Ochnaceae**  *Cespedesia spathulata* (Ruiz & Pav.) Planch. | 1 | 1 | 0,6 | 0,8 | 0,6 | 2,1 |
| *Ouratea coccinea* Engl. | 1 | 1 | 0,6 | 0,8 | 0,2 | 1,7 |
| **Phyllanthaceae**  *Hieronyma alchorneoides* Allemão | 2 | 2 | 1,2 | 1,7 | 1,7 | 4,6 |
| **Polygonaceae.**  *Triplaris surinamensis* Cham. | 14 | 3 | 8,2 | 8,5 | 13,5 | 30,2 |
| **Rubiaceae Juss.**  *Palicourea marcgravii* A.St.-Hil. | 4 | 1 | 2,4 | 1,7 | 0,1 | 4,1 |
| **Urticaceae Juss.**  *Cecropia latiloba* Miq. | 2 | 1 | 1,2 | 1,7 | 4,9 | 7,73 |
| **Total abundance** | 170 |  | - | - | - | - |
| IVI is calculated by the sum of relative density, relative frequency and relative dominance (basal area) of each species in all inventoried plots. | | | | | | |
